# Clinical Remission in Patients Affected by Severe Eosinophilic Asthma on Dupilumab Therapy: A Long-Term Real-Life Study

**DOI:** 10.3390/jcm13010291

**Published:** 2024-01-04

**Authors:** Carla Maria Irene Quarato, Pasquale Tondo, Donato Lacedonia, Piera Soccio, Paolo Fuso, Eugenio Sabato, Anela Hoxhallari, Maria Pia Foschino Barbaro, Giulia Scioscia

**Affiliations:** 1 Institute of Respiratory Diseases, Policlinico Universitario “Riuniti” di Foggia, 71122 Foggia, Italy; carlamariairene.quarato@gmail.com (C.M.I.Q.); mariapia.foschino@unifg.it (M.P.F.B.); 2 Department of Medical and Surgical Sciences, University of Foggia, 71122 Foggia, Italy; pasquale.tondo@unifg.it (P.T.); piera.soccio@unifg.it (P.S.);; 3 Respiratory Diseases Unit, “A. Perrino” P.O di Brindisi, 72100 Brindisi, Italy

**Keywords:** severe asthma, biologic therapy, dupilumab, clinical remission

## Abstract

**Background.** Nowadays, highly selective biological drugs offer the possibility of treating severe type 2 asthma. However, in the real-life setting, it is crucial to confirm the validity of the chosen biological treatment by evaluating the achievement of clinical remission. **Study purpose.** The main aims of this real-life study were to evaluate the efficacy of dupilumab in terms of clinical, functional, and inflammatory outcomes at 6, 12, 18, and 24 months of treatment and to estimate the percentage of patients achieving partial or complete clinical remission at 12 and 24 months of treatment. In addition, we attempted to identify whether baseline clinical characteristics of patients could be associated with clinical remission at 24 months of treatment. **Materials and methods.** In this observational prospective study, 20 outpatients with severe uncontrolled eosinophilic asthma were prescribed dupilumab and followed-up after 6, 12, 18, and 24 months of treatment. At each patient visit, **the** need for oral corticosteroids (OCS) and corticosteroid required dose, number of exacerbations during the previous year or from the previous visit, asthma control test (ACT) score, pre-bronchodilator forced expiratory volume in the 1st second (FEV_1_), fractional exhaled nitric oxide at a flow rate of 50 mL/s (FeNO_50_), and blood eosinophil count were assessed. **Results.** The number of OCS-dependent patients was reduced from 10 (50%) at baseline to 5 (25%) at one year (T12) and 2 years (T24). The average dose of OCS required by patients demonstrated a significant reduction at T12 (12.5 ± 13.75 mg vs. 2.63 ± 3.94 mg, *p* = 0.015), remaining significant even at T24 (12.5 ± 13.75 mg vs. 2.63 ± 3.94 mg, *p* = 0.016). The number of exacerbators showed a statistically significant decrease at T24 (10 patients, 50% vs. 3 patients, 15%, *p* = 0.03). The mean number of exacerbations demonstrated a statistically significant reduction at T24 (1.45 ± 1.58 vs. 0.25 ± 0.43, *p* = 0.02). The ACT score improved in a statistically significant manner at T12 (15.30 ± 4.16 vs. 21.40 ± 2.35, *p* < 0.0001), improving further at T24 (15.30 ± 4.16 vs. 22.10 ± 2.59, *p* < 0.0001). The improvement in pre-bronchodilator FEV_1_ values reached statistical significance at T24 (79.5 ± 14.4 vs. 87.7 ± 13.8, *p* = 0.03). The reduction in flow at the level of the small airways (FEF_25–75%_) also demonstrated an improvement, although it did not reach statistical significance either at T12 or T24. A total of 11 patients (55%) showed clinical remission at T12 (6 complete + 5 partial) and 12 patients (60%) reached clinical remission at T24 (9 complete + 3 partial). Only obesity was associated with a negative odds ratio (OR) for achieving clinical remission at T24 (OR: 0.03, 95% CI: 0.002–0.41, *p* = 0.004). No other statistically significant differences in baseline characteristics emerged between patients who reached clinical remission at T24 and the group of patients who did not achieve this outcome. **Conclusion.** Dupilumab appears to be an effective drug in promoting achievement of clinical remission in patients with severe uncontrolled eosinophilic asthma. The achievement of clinical remission should be continuously evaluated during treatment. Further studies are needed to clarify whether certain baseline clinical characteristics can help predict dupilumab favorable outcomes.

## 1. Introduction

Around 339 million people worldwide are affected by asthma, with approximately 5–10% suffering from uncontrolled or severe asthma [1,2]. Suboptimal asthma control can have a significant impact on patients, resulting in an increase in hospitalization and mortality, a decrease in quality of life, and a higher cost of health care [2].

Severe asthma is characterized by asthma that remains uncontrolled despite adherence to maximally optimized therapy and treatment of contributing factors or worsens when high-dose treatment is reduced [3]. Furthermore, patients with severe asthma exhibit low lung function and an elevated number of leukocytes in the blood, particularly eosinophils and neutrophils [4]. The immune dysregulation in severe asthma is highly heterogeneous, implying different inflammatory phenotypes [5]. Nowadays, targeting specific mediators involved in the inflammatory cascade using highly selective biological drugs offers the possibility of achieving optimal disease control in severe type 2 asthma. 

When severe asthma is diagnosed, referral to a specialist center for phenotypic study and evaluation of eligibility for additional therapy with biological drugs is therefore recommended [3]. Once a specific biological drug has been chosen, it is also crucial to confirm the validity of the therapeutic strategy by evaluating the eventual achievement of clinical remission. 

In accordance with a recent Delphi Consensus [6], clinical remission in asthma is considered “complete” when there is no longer a requirement for oral corticosteroids, and all three of the following criteria are fulfilled: the absence of asthma symptoms, the absence of asthma exacerbations, and stable lung function. On the other hand, when oral corticosteroids are no longer needed and at least two of the three criteria mentioned above are met, a “partial” clinical remission is achieved. Another expert consensus by Menzies-Gow at al. [7] suggested a more general concept of “complete remission”, involving the objective resolution of asthma-related inflammation in addition to clinical remission.

Dupilumab is a human monoclonal antibody that inhibits interleukin 4 (IL-4) and interleukin 13 (IL-13) signal transduction by binding to the alpha subunit of the interleukin IL-4 receptor (which is common to both the IL-4 and IL-13 receptors) [8,9]. 

The primary objective of this long-term real-life study was to assess the effectiveness of dupilumab in terms of clinical, inflammatory, and functional outcomes at 6, 12, 18, and 24 months of treatment in a population of patients suffering from uncontrolled, severe, type 2 asthma and to assess the clinical effectiveness of this biological drug in achieving partial or complete clinical remission at 12 and 24 months of treatment. In addition, we attempted to identify which baseline clinical characteristics of patients with severe asthma could be associated with achieving clinical remission during dupilumab treatment.

## 2. Materials and Methods

In this two-year retrospective observational study, we enrolled 20 patients from the outpatients’ clinics of the University Hospital of Policlinico of Foggia and Hospital Perrino of Brindisi (Italy) suffering from severe uncontrolled eosinophilic asthma who were selected for a therapeutic step-up with the biological drug dupilumab. 

The diagnosis of asthma was achieved by spirometry with a positive bronchodilator response or confirmatory methacholine challenge testing. Adult-onset asthma was regarded as self-reported symptoms of asthma or initiation of drug therapy for asthma at an age greater than 20 years. Inclusion criteria encompassed individuals aged 18 years or older with asthma that was not adequately controlled despite the use of maximum daily inhaled treatment and another controller (as per STEP 4–5 of GINA guidelines) or oral corticosteroid (OCS) therapy for a minimum of 6 months in the preceding year, along with peripheral blood eosinophil counts exceeding 150 eosinophils/mm^3^. Participants were excluded from the study if they demonstrated incomplete adherence to their prescribed asthma maintenance therapy or exhibited improper inhaler technique. All enrolled patients provided written informed consent. The Ethics Committee of the Policlinico Riuniti of Foggia approved the study (Institutional Review Board approval N°17/CE/12 June 2014), which complied with the principles of the Declaration of Helsinki.

Dupilumab was administered at the approved dosage of 600 mg (two injections of 300 mg each) as an initial dose, followed by 300 mg every other week, delivered via subcutaneous injection. This prescription approach was employed because patients were dependent on oral corticosteroids (OCS) or had comorbidities such as nasal polyposis or atopic dermatitis.

All 20 participants in the study underwent assessments at the beginning and subsequently at 6, 12, 18, and 24 months after initiating treatment. During each visit, the following parameters were documented and assessed comparatively: need for oral corticosteroids (OCS) and corticosteroid required dose, number of exacerbations during the previous year or from the previous visit, asthma control test (ACT) score, pre-bronchodilator forced expiratory volume in 1st second (FEV1), fractional exhaled nitric oxide at a flow rate of 50 mL/s (FeNO_50_), and blood eosinophil count.

### 2.1. Asthma Control Test

The Asthma Control Test (ACT) comprises inquiries related to symptoms and the utilization of rescue medications, supplemented by a self-evaluation test conducted by the patient to gauge their level of control over asthma. Scores on the ACT range from 5 to 25, with higher scores indicating better control. Asthma is categorized as well-controlled with scores between 20 and 25, poorly controlled with scores between 16 and 20, and uncontrolled with scores between 5 and 15. The minimal clinically important difference is defined as a three-point change in score [10].

### 2.2. Pulmonary Function Tests 

Pulmonary function tests (PFTs) were conducted with a calibrated spirometer (Sensormedics, Milan, Italy). The measurements of FEV1, FVC, and FEF_25–75%_ were acquired through maximally forced inspiratory and expiratory maneuvers. Each result, representing the best outcome from three reproducible measurements, was expressed as a percentage of the predicted values calculated using the equations developed by Quanjer and Stocks [11,12]. The presence of small airway flow limitation was determined when FEF_25–75%_ values were below 65% of the predicted values [13].

### 2.3. Inflammatory Characterization

All of the enrolled patients underwent a peripheral blood sample to evaluate the leukocyte formula and specifically the blood count and percentage of eosinophils. The measurement of FeNO_50_ was used as a marker of airway inflammation. A FeNO_50_ level ≥ 25 parts per billion (ppb) was considered a predictor of a response to dupilumab treatment [14]. 

### 2.4. Definition of Clinical Remission

To conduct this analysis, we used a definition of clinical remission based on the following four criteria:

1. lack of need to use OCS;
2. lack of exacerbations;
3. achievement of ACT score ≥ 20;
4. achievement of a percentage of pre-bd FEV1 ≥ 80% of the predicted.

To define a complete clinical remission, it was necessary for patients to satisfy all four criteria, whereas partial clinical remission was identified by the presence of at least three of the four criteria (one of which had to necessarily be the lack of use of OCS) [6].

### 2.5. Statistical Analysis 

Numerical values are presented as mean ± standard deviation (SD), and categorical data are described as number (*n*) and percentage (%) of individuals. Statistical analysis involved the use of Student’s *t*-test for continuous variables and the Fisher’s exact test for binary variables. The Odds Ratio, with a 95% confidence interval, was computed to assess the association between patients’ baseline clinical characteristics (T0) and the attainment of clinical remission at the two-year mark (T24). Statistical significance was set at *p* < 0.05. Data analysis was performed using GraphPad software (version 8, GraphPad Software Inc., San Diego, CA, USA).

## 3. Results 

### 3.1. Characteristics of the Population at Baseline (T0)

The average age of the patients was 52.25 ± 10.35 years, with a clear predominance of females (80%). The mean age of asthma onset was 40.68 ± 11.52 years, with 3 patients (15%) reporting a childhood-onset and 17 (85%) an adult-onset. Two patients (10%) were active smokers, 8 patients (40%) were former smokers, and 10 patients (50%) had never smoked. The mean BMI value was 30.04 ± 7.60. Positivity for atopy at the Prick Test was documented in 80% of cases, with an average value for total IgE of 825.40 ± 661.94. On blood tests, the mean value of the eosinophil count at baseline was 345.50 ± 194.15 cells/µL, whereas the mean FeNO_50_ level was 27.94 ± 17.72 ppb.

As regards comorbidities, 8 patients (40%) were affected by gastroesophageal reflux, 5 patients (25%) presented with nasal polyposis, 3 patients (15%) were treated simultaneously for Obstructive Sleep Apnea Syndrome (OSAS) by nocturnal ventilation, 3 patients (15%) were suffering from anxiety and depression, 2 patients (10%) had atopic dermatitis and 2 (10%) patients were suffering from osteoporosis.

At baseline (T0), 10 patients (50%) complained of exacerbations during the previous year, for an average of 1.45 ± 1.58 exacerbations/year (min: 1–max: 7). Of them, 5 patients (50%) had been hospitalized due to asthma exacerbation. Ten patients (50%) were forced to take oral corticosteroids to maintain disease control, with an average dosage of 12.5 ± 13.75 mg. The average ACT score relative to the total number of patients in the sample (average ACT 15.30 ± 4.16) denoted lack of disease control (ACT < 20 points). As regards the pulmonary function indices of the entire sample, the average FEV_1_ at baseline was approximately 79.5 ± 14.4, with an average FEV_1_/FVC ratio of 68.35 ± 9.09, thus presenting an overall moderate degree of obstruction. Furthermore, 11 patients (55%) presented a reduction in flow relating to the small airways (FEF_25–75%_), with an average value of 62.75 ± 23.82 (Table 1).

### 3.2. OCS-Dependent Patients and Average OCS Dose Required

**Figure 1:**
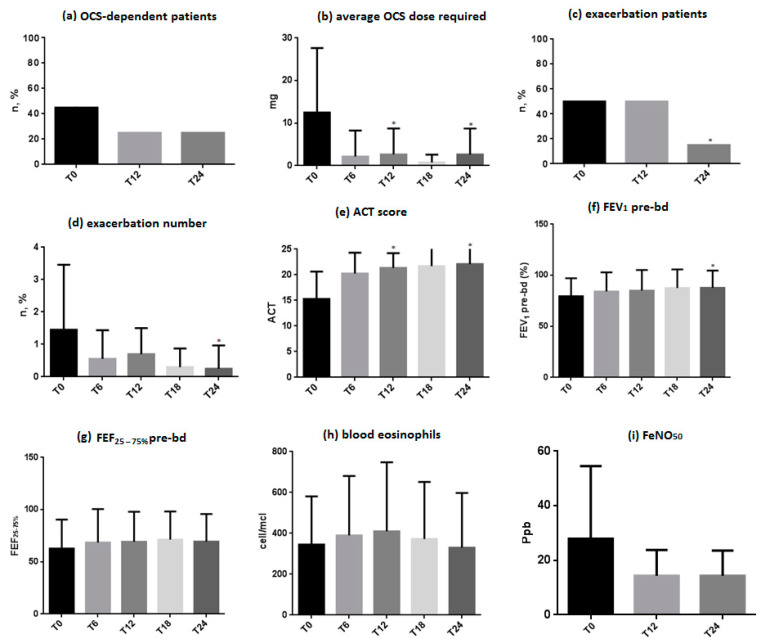
Clinical outcomes: (**a**) OCS-dependent patients; (**b**) average OCS dose required; (**c**) exacerbation patients; (**d**) exacerbation number; (**e**) ACT score; (**f**) FEV_1_ pre-bd; (**g**) FEF_25–75%_ pre-bd; (**h**) blood eosinophils; (**i**) FeNO_50_. Statistically significant differences are highlighted with *.

The number of patients dependent on OCS was reduced from 10 (50%) at baseline to 5 (25%) at one year (T12) and 2 years (T24), a difference which, although not statistically significant (*p* = 0.11), has proven to be maintained over time (Figure 1a). 

The average dose of OCS required by patients demonstrated a significant reduction at one year (12.5 ± 13.75 mg vs. 2.63 ± 3.94 mg, *p* = 0.015). This reduction remained significant even at 2 years of treatment (12.5 ± 13.75 mg vs. 2.63 ± 3.94 mg, *p* = 0.016) (Figure 1b).

### 3.3. Exacerbation Patients and Number of Exacerbations

The number of patients presenting with exacerbations remained unchanged after 1 year, but was shown to decrease, in a statistically significant manner, at 2 years of treatment (10 patients, 50% vs. 3 patients, 15%, *p* = 0.03) (Figure 1c).

The mean number of exacerbations demonstrated a progressive reduction at 1 year (1.45 ± 1.58 vs. 0.70 ± 0.70, *p* = 0.10), reaching statistical significance at 2 years of treatment (1.45 ± 1.58 vs. 0.25 ± 0.43, *p* = 0.02) (Figure 1d). None of the patients required hospitalization due to asthma exacerbations during treatment with dupilumab.

### 3.4. ACT Score

The ACT score progressively improved. This improvement was shown to reach statistical significance at one year (15.30 ± 4.16 vs. 21.40 ± 2.35, *p* < 0.0001), and then improved further at 2 years (15.30 ± 4.16 vs. 22.10 ± 2.59, *p* < 0.0001) (Figure 1e).

### 3.5. Respiratory Function 

Pre-bronchodilation FEV_1_ values showed a progressive improvement in respiratory function, which was not significant at 1 year (79.5 ± 14.4 vs. 85.05 ± 16.14, *p* = 0.06), but reached statistical significance at 2 years of treatment (79.5 ± 14.4 vs. 87.7 ± 13.8, *p* = 0.03) (Figure 1f).

The reduction in flow at the level of the small airways (FEF_25–75%_) also demonstrated an improvement, although not reaching statistical significance either at 1 year (62.75 ± 23.82 vs. 69.25 ± 22.58, *p* = 0.06) or at 2 years of treatment (62.75 ± 23.82 vs. 69.5 ± 20.65, *p* = 0.13) (Figure 1g).

### 3.6. Blood Eosinophils

The mean value of blood eosinophils was shown to increase at 1 year (345.50 ± 194.15 cells/µL vs. 409.80 ± 225.8 cells/µL, *p* = 0.3) and again reached values similar to baseline at 2 years (345.50 ± 194.15 cells/µL vs. 330.10 ± 218.00 cells/µL, *p* = 0.8) (Figure 1h).

### 3.7. FeNO_50_ Levels

FeNO_50_ levels showed a progressive reduction from baseline, although not reaching statistical significance either at 1 year (27.94 ± 17.72 ppb vs. 14.37 ± 7.38 ppb, *p* = 0.18) or at 2 years of treatment (27.94 ± 17.72 ppb vs. 14.36 ± 7.74 ppb, *p* = 0.10) (Figure 1i).

### 3.8. Clinical Remission

Based on the given definition, 6 out of 20 patients (30%) achieved complete clinical remission 1 year after starting treatment with dupilumab, while 9 patients (45%) showed complete clinical remission after 2 years of treatment (*p* = 0.51). Five patients (25%) achieved partial clinical remission at T12, while 3 patients (15%) were in partial clinical remission at T24 (*p* = 0.69).

Among the 6 patients who achieved complete clinical remission at 1 year, 4 (67%) maintained the results obtained after 2 years, while the other 2 patients (33%) did not fully meet the definition for the occurrence of an exacerbation. However, among these 2 patients, only 1 experienced an exacerbation such as to require the use of OCS; the other patient remained in a condition of partial clinical remission. Among the 9 patients who achieved complete clinical remission after two years, 4 (44%) had reached the state of partial clinical remission already 1 year after starting dupilumab. One patient (5%) achieved a state of partial clinical remission at T12, which remained so at T24. Finally, the state of partial clinical remission was achieved by an additional patient after 2 years of treatment.

**Figure 2:**
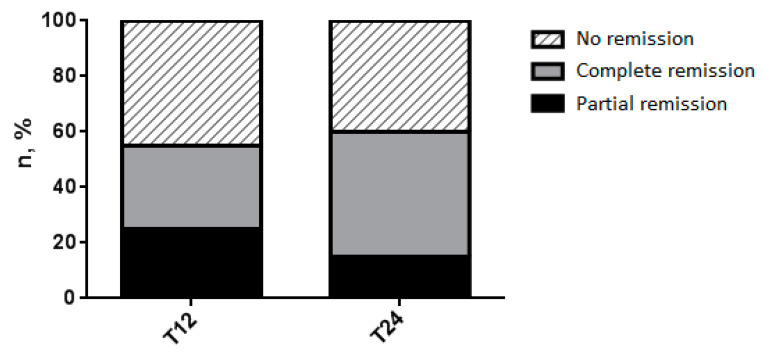
Patients reaching clinical remission.

Adding the two definitions together, a total of 11 patients (55%) showed clinical remission (6 complete + 5 partial) at T12 and 12 patients (60%) reached clinical remission (9 complete + 3 partial) at T24 (*p* = 1.00) (Figure 2).

### 3.9. Analysis of the Characteristics Shown at Baseline by Patients in Clinical Remission (Partial + Complete) at T24

**Figure 3:**
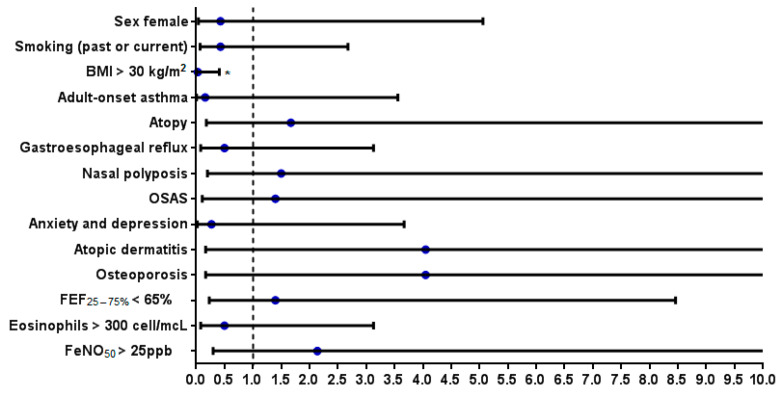
OR of clinical characteristics for achieving clinical remission at T24. Statistically significant differences are highlighted with *.

Analyzing the clinical characteristics presented at baseline of the patients who achieved clinical remission (partial and/or complete) at T24, no statistically significant differences emerged compared to the group of patients who did not achieve this outcome, except for obesity only (BMI > 30 kg/m^2^) which would appear to be a negative predictive factor for the success of the treatment (OR: 0.03, 95% CI: 0.002–0.41, *p* = 0.004) (Table 2, Figure 3).

## 4. Discussion

Our study involved 20 patients diagnosed with severe uncontrolled asthma type 2 (mean ACT: 15.30 ± 4.16) and an average eosinophil count of 345.50 ± 194.15 cells/µL who were selected for a therapeutic step-up with the biological drug dupilumab, since they had had at least two asthma exacerbations or one hospitalization due to asthma exacerbations in the previous year (50% of patients with a mean number of 1.45 ± 1.58 exacerbations/year) or were dependent on oral corticosteroids (50% of patients, with a mean daily OCS dose of 12.5 ± 13.75 mg) before starting treatment.

According to the results of our study, treatment with dupilumab resulted in a reduction in the number of OCS-dependent patients from 10 (50%) at baseline to 5 (25%) at T12 and at T24, with a significant reduction in the average OCS dose necessary to maintain asthma control after 1 year of treatment (12.5 ± 13.75 mg vs. 2.63 ± 3.94 mg, *p* = 0.015), and which was maintained even after 2 years (12.5 ± 13.75 mg vs. 2.63 ± 3.94 mg, *p* = 0.016). These results are consistent with those previously observed in the literature. It is worth mentioning the phase 3 VENTURE study, in which dupilumab administered as subcutaneous injection at 300 mg dosage every other week significantly reduced OCS dose versus placebo in patients with OCS-dependent severe asthma after 24 weeks of treatment [15]. Additionally, the 52-week phase 3 QUEST study demonstrated the effectiveness of dupilumab, administered as a subcutaneous injection at 200 mg and 300 mg dosages every other week versus placebo, in improving the ACT score, the exacerbation rate, and lung function in patients with moderate to severe asthma [14]. In addition, Dupin et al. [16] showed that dupilumab was able to improve asthma control and respiratory function, as well as to reduce asthma exacerbation and daily OCS dose, in 64 patients with severe asthma after 12 months of treatment in a real-life setting. The same favorable clinical outcomes were confirmed by the 1-year real-life experience reported by Campisi et al. [17]. Similarly, in our study, the ACT score progressively improved, reaching a statistically significant increase after 1 year of treatment (15.30 ± 4.16 vs. 21.40 ± 2.35, *p* < 0.0001), and then improving further after 2 years (15.30 ± 4.16 vs. 22.10 ± 2.59, *p* < 0.0001). On the other hand, albeit that we recorded a progressive reduction in the number of exacerbations (1.45 ± 1.58 vs. 0.70 ± 0.70 at T12), it reached statistical significance only after 2 years of therapy (1.45 ± 1.58 vs. 0.25 ± 0.43, *p* = 0.02) along with a statistically significant reduction in number of patients experiencing exacerbations at T24 (10 patients, 50% vs. 3 patients, 15%, *p* = 0.03). Notably, none of the patients reported hospitalizations due to asthma exacerbations while receiving dupilumab. Furthermore, dupilumab also acted on respiratory function by contributing to an increase in the pre-bronchodilation FEV_1_ values that, while not statistically significant at T12 (79.5 ± 14.4 vs. 85.05 ± 16.14, *p* = 0.06), did reach statistical significance at T24 (79.5 ± 14.4 vs. 87.7 ± 13.8, *p* = 0.03).

Finally, in our study, treatment with dupilumab showed an increase in the number of blood eosinophils at T12 (345.50 ± 194.15 cells/µL vs. 409.80 ± 225.8 cells/µL, *p* = 0.30). However, blood eosinophil counts returned to baseline values at 2 years (345.50 ± 194.15 cells/µL vs. 330.10 ± 218.00 cells/µL, *p* = 0.80). Transient eosinophilia was also documented in pivotal phase 3 studies [14,15]. This effect could be related to the mechanism of action of dupilumab. Indeed, the blockade of IL-4 signaling induces a depletion of eosinophils in the lung, caused by a reduction in vascular cell adhesion molecule-1 (VCAM-1) expressed by endothelial cells and the consequent blockage of pulmonary chemotaxis, while circulating eosinophils remain unchanged and may transiently increase due to the lack of pulmonary sequestration [18]. The transient increase in blood eosinophil counts during the first year of dupilumab therapy in our study was not associated with clinical consequence and did not affect treatment efficacy, as shown by the concomitant reduction in OCS use and asthma exacerbation and the lack of hospitalizations. However, the apparent discrepancy between the important observed variation in eosinophil counts and the lack of statistical significance could also be due to our small sample size. Considering that high levels of blood eosinophils may be masked by chronic OCS use and that in OCS-dependent severe asthma patients an underlying (ANCA-negative) eosinophilic granulomatosis with polyangiitis (EGPA) has always to be suspected, a serial assessment of blood eosinophil counts during treatment with an anti-IL-4/IL-5 biologic seems advisable. In this regard, Edger et al. [19] suggested stopping dupilumab and shifting to an anti-IL-5 treatment if eosinophils rise to more than 1000 cells/µL and/or asthma symptoms worsen. Otherwise, the degree of airway inflammation has been shown to decline after 1 and 2 years of treatment, albeit not in a statistically significant manner, as evidenced by the FeNO_50_ levels in our patients. Nonetheless, baseline FeNO_50_ levels may have been reduced by OCS use or may have been biased by some comorbidity conditions, such as obesity. 

We also analyzed the effectiveness of dupilumab after 1 and 2 years of treatment, in terms of the percentage of patients who managed to undergo partial and/or complete clinical remission, according to the definition given by Canonica et al. [6]. A total of 11 participants (55%) out of 20 enrolled patients achieved clinical remission at T12 (of which 6 were complete and 5 were partial), while 12 (60%) were in clinical remission after 2 years of treatment with this biological drug (of which 9 were complete and 3 were partial). The data is in line with a recent study by Pavord et al. [20] which demonstrated the achievement of clinical remission in patients suffering from severe uncontrolled type 2 asthma after biological therapy with dupilumab. 

An interesting fact that emerged from our study is that the transition from a state of partial to complete remission appears very dynamic, with the possibility of oscillating between these two conditions from one year of treatment to another. 

The most important data that should guide us in judging a certain biological therapy as appropriate seems to be the lack of need of OCS use. The possibility of interrupting therapy with OCS is extremely relevant, since OCS, if used both continuously and intermittently, entails an increased risk of adverse events in subjects with asthma. Among these, a reduction in immune defenses with the risk of hospitalization or access to the emergency room is certainly the most dangerous. Furthermore, the use of OCS increases the risk of developing various comorbidities, such as cataracts, pneumonia, type 2 diabetes, cardiovascular disease, renal failure, and osteoporosis, and increases the mortality rate [21]. 

On the other hand, the parameter that is most likely to lead to a failure to meet requirements for assigning a complete remission status seems to be presence of exacerbations, although these do not require the use of OCS. Indeed, the failure to assign a status of complete remission may also be influenced by the lack of a clear definition of “asthma exacerbation”. As result, in a real-life setting it is easy to mistake a simple bacterial upper respiratory infection requiring antibiotics for an exacerbation, even without the presence of an actual bronchial inflammation or the worsening of respiratory symptoms. Therefore, to improve control of the disease and ensure that a state of complete remission is achieved, we can speculate on the usefulness of advising the patient to undergo the recommended vaccinations [22] or preventive immunoprophylaxis with bacterial lysates [23].

The use of biological drugs in the treatment of a chronic disease such as severe asthma is burdened by high costs. Hence, there is an urgent need to identify factors predictive of an adequate clinical response for each biological agent. 

By specifically binding to the IL-4Rα receptor subunit, dupilumab blocks both IL-4 and IL-13 signaling, thereby limiting the production of serum IgE and suppressing both the upstream and downstream inflammatory type 2 cascade. This mechanism of action explains why dupilumab, in addition to treating severe type 2 asthma, can have a therapeutic role in several type 2 disorders, such as chronic rhinosinusitis with nasal polyposis, atopic dermatitis, eosinophilic esophagitis, and prurigo nodularis [24]. In a *post-hoc* analysis of the phase 3 QUEST study [25], dupilumab was shown to be equally effective in allergic and non-allergic moderate-to-severe asthmatics. In the real-life study by Pelaia et al. [26], dupilumab was shown to induce a positive impact on many clinical and functional parameters in patients with severe asthma and nasal polyposis as soon as 4 weeks after the first administration. Fomina et al. [27] retrospectively observed, within the first 6 months of treatment with dupilumab, an improvement in asthma control and respiratory function, as well as a reduction in the frequency of exacerbations associated with the use of OCS in 115 patients with uncontrolled severe asthma, regardless of the presence or absence of atopic dermatitis. Chan et al. [28] reported significant improvements in oscillometry outcomes in conjunction with significant improvements in asthma control in 16 patients with uncontrolled severe asthma and concomitant oscillometry-defined small airways dysfunction (SAD) after a mean of 4.5 months of treatment with dupilumab. In this regard, it was speculated that the blockage of IL-4 and IL-13 signaling may limit the phenomenon of bronchial smooth muscle contraction and hypertrophy which leads, at least in part, to the airway remodeling process [28]. A blood eosinophil count ≥ 300 cells/µL and a FeNO_50_ level ≥ 25 ppb are known predictive factors of a positive response to treatment with dupilumab [14]. Both these biomarkers have been associated with an increased risk for asthma exacerbations [29].

In our real-life experience, analyzing the baseline clinical characteristics of the patients who achieved clinical remission (partial and/or complete) after 2 years of treatment, no statistically significant differences emerged compared to the group of patients who did not achieve this outcome, with the only exception of obesity (BMI > 30 kg/m^2^), which would appear to be a negative predictive factor for the success of the treatment (OR: 0.03, 95% CI: 0.002–0.41, *p* = 0.004). Although without reaching statistical significance, a positive OR for achieving clinical remission was calculated for factors such as atopy, nasal polyposis, OSAS, atopic dermatitis, osteoporosis, small airway flow limitation (FEF_25–75%_ < 65%), and FeNO_50_ >25 ppb. It should be noted that all patients with atopic dermatitis and osteoporosis (the latter, presumably heavy users of OCS) achieved clinical remission at T24.

Observational studies have shown that obesity is an independent risk factor for increased asthma severity, worse disease control, lower quality of life, and a higher risk of exacerbations [30]. Several mechanisms have been proposed to explain the role of obesity in asthma, including a combination of mechanical and inflammatory factors. Excessive accumulation of fat in the thoracic and abdominal cavities may lead to parenchymal compression and reduction of lung volumes, with more marked effects on functional residual capacity (FRC) and expiratory reserve volume (ERV) [31]. Considering that alveolar tension pulling the lung airways open is lowered at low lung volumes, this may contribute to facilitate airway expiratory collapse in obese patients [32]. In addition, obesity is associated with a state of chronic low-grade inflammation due to a relatively poor blood supply in hypertrophic adipose tissue, leading to hypoxic suffering and death of some adipocytes. The consequent increased production of pro-inflammatory cytokines by the adipose tissue induces insulin-resistance and further systemic inflammation with adipose tissue dysfunction [33]. This systemic state of low-grade inflammation could potentially influence and worsen airway inflammation in obese asthmatics. For example, an increase in airway oxidative stress in obese asthmatics has been related to a reduction in the bioavailability of arginine, which is a substrate for the production of nitric oxide (NO). This could be the reason why obesity in late-onset asthma is frequently characterized by normal or low values of exhaled nitric oxide (FeNO) [34]. As NO is an endogenous bronchodilator, reduced NO production may contribute to airway disease in obesity [34,35]. Similarly, the increased production of inflammatory cytokines in obesity has been related to reduced induction of mitogen-activated kinase phosphatase-1 (MKP-1) by glucocorticoid, which is a signaling protein that plays an important role in steroid responses. This could explain why obese asthmatics respond less to standard control treatments with inhaled corticosteroids (ICS) [36]. In regard to the negative impact of obesity on dupilumab treatment, a recent pharmacokinetic analysis by Zhang et al. [37] highlighted a lower drug distribution in obese asthma patients compared to normal-weight patients. However, these authors concluded that weight-based dose adjustments are unnecessary due to a limited difference in efficacy and safety between different weight categories [37]. On the other hand, asthma itself and chronic OCS use may be predisposing factors leading to obesity [38,39]. In this regard, it would be interesting to evaluate in future studies whether dupilumab, by reducing the average OCS dose necessary to maintain asthma control, is also able to reduce the BMI of treated patients with a consequent increase in therapeutic effects over time. 

Failure to achieve statistical significance in identifying clinical factors associated with remission may have been due to the small sample size, that can also be regarded as the main limitation of our study. However, dupilumab was approved in Italy for the treatment of patients with severe asthma and type 2 inflammation only as recently as 2019. In light of this data, a number of 20 patients appears to be a fairly adequate number to evaluate the effectiveness of the treatment with this biological drug over a period of 2 years. Other limitations are quite common to other real-life studies and included the lack of randomization design and placebo control. These limits did not allow factors potentially influencing the outcomes to be properly balanced and the effectiveness of the treatment to be compared with a possible placebo effect. 

In any case, real-life settings reflect routine clinical practice and have the advantage of including in the evaluation of treatment efficacy those severe asthma patients presenting clinical characteristics and comorbidities who would have been excluded from randomized controlled trials (RCTs). That said, real-world research appears crucial for the identification of actual clinical or biological prediction factors for the path toward the goal of clinical remission with biological treatment. 

The main strengths of our study are that it allowed the assessment of dupilumab real-life effectiveness in patients with uncontrolled or OCS-dependent severe asthma in the longer term (i.e., 2 years after the first administration) in contrast to other studies in the literature and that it focused on the importance of continuously evaluating the effectiveness of a biological treatment in reaching and maintaining clinical remission for severe asthma patients. 

Despite our study limitations that could have affected the generalizability of the results and the confidence in the identified association between the patients’ clinical characteristics3e3 and achievement of clinical remission, we hope that our observations would give the opportunity to enhance attention in this field of clinical research and promote future studies that could support or refute the findings presented. Comparison between different real-life experiences and complementary results from RCTs can be an effective strategy to overcome the limitations of each study individually taken. 

## 5. Conclusions

Dupilumab appears to be an effective drug in promoting the achievement of clinical remission in patients suffering from severe uncontrolled eosinophilic asthma. The state of clinical remission seems to be a very dynamic condition that needs to be continuously evaluated throughout the treatment. 

Further studies in a larger population are needed to clarify whether certain baseline clinical characteristics can help predict dupilumab treatment outcomes, increase clinical remission rates, and potentially modify disease progression.

## Figures and Tables

**Table 1:** Characteristics of the population at baseline (T0).

Characteristics	Results (*n* = 20)
Age, years (mean ± SD)	52.25 ± 10.35
Females, *n* (%)	16 (80%)
Males, *n* (%)	4 (20%)
Current smokers, *n* (%)	2 (10%)
Past smokers, *n* (%)	8 (40%)
BMI, kg/m^2^ (mean ± SD)	30.04 ± 7.60
Age of asthma onset, years (mean ± SD)	40.68 ± 11.52
Childhood-onset asthma, *n* (%)	3 (15%)
Adult-onset asthma, *n* (%)	17 (85%)
Atopy, *n* (%)	16 (80%)
Gastroesophageal reflux, *n* (%)	8 (40%)
Nasal polyposis, *n* (%)	5 (25%)
OSAS, *n* (%)	3 (15%)
Anxiety and depression, *n* (%)	3 (15%)
Atopic dermatitis, *n* (%)	2 (10%)
Osteoporosis, *n* (%)	2 (10%)
Exacerbation patients, *n* (%)	10 (50%)
Exacerbation/year, *n* (mean ± SD)	1.45 ± 1.58
OCS-dependent patients, *n* (%)	10 (50%)
OCS dosage, mg (mean ± SD)	12.5 ± 13.75
ACT score (mean ± DS)	15.30 ± 4.16
FEV_1_/FVC (mean ± DS)	68.35 ± 9.09
FEV_1_% _pre bd_, (mean ± DS)	79.50 ± 14.40
FEV_1, Liters_, (mean ± DS)	91.15 ± 14.32
FEF_25–75%_, (mean ± DS)	62.75 ± 23.82
Eosinophils, cells/µL (mean ± DS)	345.50 ± 194.15
Eosinophils > 300 cells/µL, *n* (%)	8 (40%)
FeNO_50_, ppb (mean ± DS)	27.94 ± 17.72
FeNO_50_ > 25 ppb, *n* (%)	7 (35%)
Total IgE, kU/L (mean ± DS)	825.40 ± 661.94

Abbreviations: SD: standard deviation; BMI: body mass index; OSAS: obstructive sleep apnea syndrome; OCS: oral corticosteroids; ACT: asthma control test; FEV_1_: forced expiratory flow in 1st second; pre bd: pre-bronchodilators; FEF_25–75%_: forced expiratory flow at 25–75% of the vital capacity; FeNO_50_: fractional exhaled nitric oxide at a flow rate of 50 mL/s.

**Table 2:** Analysis of the characteristics shown at baseline of remitting patients at T24.

Characteristics at Baseline (T0)	Clinical Remission at T24 (*n* = 12)	No Remission at T24 (*n* = 8)	OR	95% CI		*p*-Value
Sex female	9 (56.3%)	7 (43.7%)	0.43	0.04	5.06	0.62
Smoking (past or current)	5 (50.0%)	5 (50.0%)	0.43	0.07	2.68	0.65
BMI > 30 kg/m^2^	1 (14.3%)	6 (85.7%)	0.03	0.002	0.41	0.004 *
Adult-onset asthma	9 (52.9%)	8 (47.1%)	0.16	0.007	3.56	0.24
Atopy	10 (62.5%)	6 (37.5%)	1.67	0.18	15.14	1.00
Gastroesophageal reflux	4 (50.0%)	4 (50.0%)	0.50	0.08	3.13	0.65
Nasal polyposis	4 (66.7%)	2 (33.3%)	1.50	0.20	11.09	1.00
OSAS	2 (66.7%)	1 (33.3%)	1.40	0.11	18.63	1.00
Anxiety and depression	1 (33.3%)	2 (66.7%)	0.27	0.02	3.67	0.54
Atopic dermatitis	2 (100.0%)	0 (0.0%)	4.05	0.17	96.26	0.49
Osteoporosis	2 (100.0%)	0 (0.0%)	4.05	0.17	96.26	0.49
FEF_25–75%_ < 65%	7 (63.6%)	4 (36.4%)	1.40	0.23	8.46	1.00
Eosinophils > 300 cell/mcL	4 (50.0%)	4 (50.0%)	0.50	0.08	3.13	0.65
FeNO_50_ > 25 ppb	5 (71.4%)	2 (28.6%)	2.14	0.30	15.36	0.64

Abbreviations: CI: confidence interval; OR: odds ratio; BMI: body mass index; OSAS: obstructive sleep apnea syndrome; FEF_25–75%_: forced expiratory flow at 25–75% of the vital capacity; FeNO_50_: fractional exhaled nitric oxide at a flow rate 50 mL/s. Statistically significant differences are highlighted with *.

## Data Availability

Data are contained within the article.
